# Supplementary material for: The Clinical and Laboratory Landscape of COVID-19 During the Initial Period of the Pandemic and at the Beginning of the Omicron Era
Source: Viruses. 2025 Mar 27;17(4):481. doi: 10.3390/v17040481 (PMC12031490; doi:10.3390/v17040481)
Supplement: Supplementary file 1 [file viruses-17-00481-s001.zip › Table S1.pdf]

Table S1. Characteristics of patients with COVID-19 depending on the gender of participants, cohort 1.

**Characteristic**	**Men (n= 22)**	**Women (n= 23)**	**P =**
day of hospitalization; Me (Q25; Q75)	58.5 (52.25; 69)	62 (60;71.5)	0.14
NLR ; reference interval 1.13-3.79 units ; Me (Q 25; Q 75)	7(6;10)	7 (5;8)	0.15
CRP; reference interval 0.00-5.00 mg-l ; Me (Q 25; Q 75)	6.95 (5.07;11.49)	5.60 (3.78;7.88)	0.28
FIBRINOGEN reference interval 2.00-4.00; Me (Q 25; Q 75)	101.74 (52.05;227.96)	85.60 (65.20;133.35)	0.46
C3; reference interval 0.9 – 1.8 g/l; Me (Q 25; Q 75)	6.67 (5.48;8.20)	5.99 (5.12;6.47)	0.07
IgG ; Me(Q25;Q75)	1.28 (1.09;2.760	1.13 (1.10;2.29)	0.80
IgM; Me (Q 25; Q 75)	0.27 (0.20;15.09)	0.26 (0.21;4.55)	0.86
TNF-α, pg / mL ; reference interval 0–8.21 pg / mL; Me (Q 25; Q 75)	0.42 (0.24;2.21)	0.62 (0.25;4.89)	0.44
Interleukin 6; reference interval 1.3–6.8 pg / mL ; Me (Q 25; Q 75)	9.82 (3.39;92.81)	13.45 (0.06;49.37)	0.36
Interferon-α, reference interval < 10 pg / mL , Me (Q 25; Q 75)	25.63 (2.53;104.35)	16.91(0.00;89.36)	0.76
day of hospitalization; Me (Q25; Q75)	19.85 (9.06;45.80)	21.51 (15.66; 34.28)	0.58
