# Supplementary material for: The Clinical and Laboratory Landscape of COVID-19 During the Initial Period of the Pandemic and at the Beginning of the Omicron Era
Source: Viruses. 2025 Mar 27;17(4):481. doi: 10.3390/v17040481 (PMC12031490; doi:10.3390/v17040481)
Supplement: Supplementary file 1 [file viruses-17-00481-s001.zip › Table S2.pdf]

Table S2. Characteristics of patients with COVID-19 depending on the gender of participants, cohort 2.

**Characteristic**	**Men (n= 27)**	**Women (n= 26)**	**P =**
day of hospitalization; Me (Q25; Q75)	4(2.5;5)	4(3;5.75)	0.77
NLR ; reference interval 1.13-3.79 units ; Me (Q 25; Q 75)	4.57(3.47;8.19)	4.59(3.29;7.37)	0.75
CRP; reference interval 0.00-5.00 mg-l ; Me (Q 25; Q 75)	84.54(24.13;119.35)	88.75(33.14;177.55)	0.21
FIBRINOGEN reference interval 2.00-4.00; Me (Q 25; Q 75)	5.19(4.51;6.34)	5.29(4.17;6.59)	0.50
C3; reference interval 0.9 – 1.8 g/l; Me (Q 25; Q 75)	8.68(5.29;11.24)	8.50(5.05;10.64)	0.78
IgG ; Me(Q25;Q75)	1.86(0.20;3.82)	2.00(0.41;3.73)	0.43
IgM ; Me (Q 25; Q 75)	4.65(0.30;7.76)	3.98(2.26;6.97)	0.76
TNF-α, pg / mL ; reference interval 0–8.21 pg / mL ; Me (Q 25; Q 75)	0.00(0.00;1.34)	0.14(0.00;1.09)	0.62
Interleukin 6; reference interval 1.3–6.8 pg / mL; Me (Q 25; Q 75)	11.10(3.74;33.00)	10.29(4.74;30.10)	0.91
Interferon-α, reference interval < 10 pg / mL , Me (Q 25; Q 75)	0.00(0.00;2.49)	0.00(0.00;0.56)	0.42
