# Supplementary material for: The Clinical and Laboratory Landscape of COVID-19 During the Initial Period of the Pandemic and at the Beginning of the Omicron Era
Source: Viruses. 2025 Mar 27;17(4):481. doi: 10.3390/v17040481 (PMC12031490; doi:10.3390/v17040481)
Supplement: Supplementary file 1 [file viruses-17-00481-s001.zip › Table S3.pdf]

Table S3. Characteristics of patients with COVID-19 depending on the age of participants, cohort 1.

**Characteristic**	**Under 65 (n= 28)**	**65 and older (n= 17)**	**P =**
Day of hospitalization; Me (Q25; Q75)	7.00(5.00;8.75)	7(6;9)	0.41
NLR; reference interval 1.13-3.79 units ; Me (Q 25; Q 75)	5.89(4.13;10.47)	6.21(5.01;7.90)	0.54
CRP; reference interval 0.00-5.00 mg-l ; Me (Q 25; Q 75)	86.10(45.43;170.33)	106.50(65.60;189.50)	0.58
FIBRINOGEN reference interval 2.00-4.00; Me (Q 25; Q 75)	6.39(5.45;7.06)	6.07(5.19;6.67)	0.23
C3; reference interval 0.9 – 1.8 g/l; Me (Q 25; Q 75)	0.32(0.20;11.93)	0.24(0.21;2.75)	0.69
IgG ; Me(Q25;Q75)	0.45(0.26;4.73)	0.39(0.24;2.06)	0.62
IgM ; Me (Q 25; Q 75)	1.11(1.09;1.65)	1.87(1.10;3.04)	0.14
TNF-α, pg / mL ; reference interval 0–8.21 pg / mL ; Me (Q 25; Q 75)	11.98(4.86;69.59)	12.76(0.31;53.67)	0.32
Interleukin 6; reference interval 1.3–6.8 pg / mL ; Me (Q 25; Q 75)	14.47(0.00;100.49)	30.10(13.40;96.77)	0.35
Interferon-α, reference interval < 10 pg / mL , Me (Q 25; Q 75)	21.45 (14.48 ;50.99)	18.55(13.37;32.75)	0.34
