# Supplementary material for: The Clinical and Laboratory Landscape of COVID-19 During the Initial Period of the Pandemic and at the Beginning of the Omicron Era
Source: Viruses. 2025 Mar 27;17(4):481. doi: 10.3390/v17040481 (PMC12031490; doi:10.3390/v17040481)
Supplement: Supplementary file 1 [file viruses-17-00481-s001.zip › Table S4.pdf]

Table S4. Characteristics of patients with COVID-19 depending on the age of participants, cohort 2.

| **Characteristic** | **Under 65 (n= 15)** | **65 and older (n= 38)** | **P =** |
|---|---|---|---|
| day of hospitalization; Me (Q25; Q75) | 4 (3;5.5) | 4 (3;5) | 0.74 |
| NLR; reference interval 1.13-3.79 units; Me (Q 25; Q 75) | 4.53 (3.99;5.19) | 4.98 (2.81;8.43) | 0.91 |
| CRP; reference interval 0.00-5.00 mg-l ; Me (Q 25; Q 75) | 118.98 (30.6;185.65) | 79 (24.21;112.3) | 0.20 |
| Fibrinogen reference interval 2.00-4.00; Me (Q 25; Q 75) | 5.82 (4.33;7.14) | 5.19 (4.33;5.91) | 0.27 |
| C3; reference interval 0.9 – 1.8 g/l; Me (Q 25; Q 75) | 9.13(4.34;12.29) | 8.48 (5.49;10.26) | 0.58 |
| IgG; Me(Q 25;Q75) | 2.56 (0.74;4.24) | 1.17 (0.21; 3.17) | 0.15 |
| IgM; Me (Q 25; Q 75) | 4.97 (1.97;7.71) | 3.41 (0.33; 7.53) | 0.38 |
| TNF-α, pg / mL; reference interval 0–8.21 pg / mL; Me (Q 25; Q 75) | 0 (0;0.3) | 0.27 (0;2.24) | 0.04 |
| Interleukin 6; reference interval 1.3–6.8 pg / mL; Me (Q 25; Q 75) | 5.98 (2.73;13.20) | 17.18 (8.54;39.22) | 0.0013 |
| Interferon-α, reference interval < 10 pg / mL, Me (Q 25; Q 75) | 0(0;0) | 0 (0; 2.20) | 0.08 |
