# Supplementary material for: The Clinical and Laboratory Landscape of COVID-19 During the Initial Period of the Pandemic and at the Beginning of the Omicron Era
Source: Viruses. 2025 Mar 27;17(4):481. doi: 10.3390/v17040481 (PMC12031490; doi:10.3390/v17040481)
Supplement: Supplementary file 1 [file viruses-17-00481-s001.zip › Table S5.pdf]

Table S5. Characteristics of patients with COVID-19 depending on the presence of vaccination, for 2021.

**Patients**	**No SARS-CoV-2 vaccination (n= 16)**	**SARS-CoV-2 vaccination (n= 37)**	**P =**
Age; Me (Q25; Q75)	67.5 (53.5;76.25)	67 (59;75)	0.82
day of hospitalization; Me (Q25; Q75)	5(3.75;6)	4 (3;5)	0.09
Mild COVID-19	5 (31%)	14 (37.8%)	0.23
Moderate COVID-19	5 (31%)	17 (46%)	0.45
Severe COVID-19	6 (38%)	6 (16.2%)	0.09
NLR; reference interval 1.13-3.79 units; Me (Q 25; Q 75)	4.82 (3.93;8.6)	4.53 (3;8.12)	0.32
CRP; reference interval 0.00-5.00 mg-l ; Me (Q 25; Q 75)	90.75 (30.5;188.28)	85.08 (27.59;157.85)	0.55
Fibrinogen reference interval 2.00-4.00; Me (Q 25; Q 75)	5.18 (4.5;6.68)	5.19 (4.31;6.35)	0.76
C3; reference interval 0.9 – 1.8 g/l; Me (Q 25; Q 75)	9.01 (4.52;10.62)	8.34 (5.94;11.33)	0.61
IgG; Me(Q 25;Q75)	1.47 (0.26;3.9)	1.96 (0.27;3.4)	0.64
IgM; Me (Q 25; Q 75)	4.62 (1.2;9.01)	3.87 (1.16;7.51)	0.56
TNF-α, pg / mL; reference interval 0–8.21 pg / mL; Me (Q 25; Q 75)	0 (0;2.24)	0 (0;1.09)	0.72
Interleukin 6; reference interval 1.3–6.8 pg / mL; Me (Q 25; Q 75)	6.18 (3.23;14.06)	13.82 (6.54;34.43)	0.18
Interferon-α, reference interval < 10 pg / mL, Me (Q 25; Q 75)	0.33 (0;3.7)	0 (0;0.92)	0.12
